# *Helicobacter pylori* Type IV Secretion System and Its Adhesin Subunit, CagL, Mediate Potent Inflammatory Responses in Primary Human Endothelial Cells

**DOI:** 10.3389/fcimb.2018.00022

**Published:** 2018-02-06

**Authors:** Mona Tafreshi, Jyeswei Guan, Rebecca J. Gorrell, Nicole Chew, Yue Xin, Virginie Deswaerte, Manfred Rohde, Roger J. Daly, Richard M. Peek, Brendan J. Jenkins, Elizabeth M. Davies, Terry Kwok

**Affiliations:** ^1^Infection & Immunity Program, Biomedicine Discovery Institute and Department of Biochemistry and Molecular Biology, Monash University, Clayton, VIC, Australia; ^2^Cancer Program, Biomedicine Discovery Institute and Department of Biochemistry and Molecular Biology, Monash University, Clayton, VIC, Australia; ^3^Infection & Immunity Program, Biomedicine Discovery Institute and Department of Microbiology, Monash University, Clayton, VIC, Australia; ^4^Centre for Innate Immunity and Infectious Diseases, Hudson Institute of Medical Research, Clayton, VIC, Australia; ^5^Department of Molecular Translational Science, School of Clinical Sciences, Monash University, Clayton, VIC, Australia; ^6^Helmholtz Centre for Infection Research, Central Facility for Microscopy, Braunschweig, Germany; ^7^Division of Gastroenterology, Vanderbilt University School of Medicine, Nashville, TN, United States

**Keywords:** *Helicobacter*, type IV secretion, interleukin-8, endothelial cells, inflammation, CagL, HUVECs

## Abstract

The Gram-negative bacterium, *Helicobacter pylori*, causes chronic gastritis, peptic ulcers, and gastric cancer in humans. Although the gastric epithelium is the primary site of *H. pylori* colonization, *H. pylori* can gain access to deeper tissues. Concurring with this notion, *H. pylori* has been found in the vicinity of endothelial cells in gastric submucosa. Endothelial cells play crucial roles in innate immune response, wound healing and tumorigenesis. This study examines the molecular mechanisms by which *H. pylori* interacts with and triggers inflammatory responses in endothelial cells. We observed that *H. pylori* infection of primary human endothelial cells stimulated secretion of the key inflammatory cytokines, interleukin-6 (IL-6) and interleukin-8 (IL-8). In particular, IL-8, a potent chemokine and angiogenic factor, was secreted by *H. pylori*-infected endothelial cells to levels ~10- to 20-fold higher than that typically observed in *H. pylori*-infected gastric epithelial cells. These inflammatory responses were triggered by the *H. pylori* type IV secretion system (T4SS) and the T4SS-associated adhesin CagL, but not the translocation substrate CagA. Moreover, in contrast to integrin α_5_β_1_ playing an essential role in IL-8 induction by *H. pylori* upon infection of gastric epithelial cells, both integrin α_5_β_1_ and integrin α_v_β_3_ were dispensable for IL-8 induction in *H. pylori*-infected endothelial cells. However, epidermal growth factor receptor (EGFR) is crucial for mediating the potent *H. pylori*-induced IL-8 response in endothelial cells. This study reveals a novel mechanism by which the *H. pylori* T4SS and its adhesin subunit, CagL, may contribute to *H. pylori* pathogenesis by stimulating the endothelial innate immune responses, while highlighting EGFR as a potential therapeutic target for controlling *H. pylori-*induced inflammation.

## Introduction

The microaerophilic Gram-negative bacterium, *Helicobacter pylori*, causes chronic gastritis, peptic ulcers, and gastric cancer in humans (Kusters et al., 2006). Almost all individuals infected by *H. pylori* develop active chronic inflammation in the stomach, characterized by infiltration of neutrophils and macrophages into the gastric mucosa (Sipponen, 1993). This inflammation is driven by various cytokines and chemokines secreted during *H. pylori* infection, including interleukin-1β, tumor necrosis factor alpha (TNF-α), IL-8, and IL-6. In particular, the level of IL-8, a potent angiogenic factor and chemoattractant, is significantly elevated in the gastric mucosa of *H. pylori*-infected patients (Crabtree, 1996; Naumann and Crabtree, 2004). The mechanism by which *H. pylori* stimulates IL-8 induction in gastric epithelial cells has been intensely studied. The *H. pylori cag* type IV secretion system (T4SS), a multi-component secretion machinery encoded by a 40-kb genetic locus named *cag* pathogenicity island (*cag*PAI), is one of the strongest known IL-8 inducing virulence factors of *H. pylori* (Fischer, 2011). Upon *H. pylori* infection of gastric epithelial cells, the *cag* T4SS stimulates IL-8 release via a multipronged mechanism that involves both the T4SS translocation substrate, CagA, and the putative T4SS adhesin and minor pilin, CagL (Gorrell et al., 2013). CagA, upon translocation by the T4SS into gastric epithelial cells, stimulates IL-8 induction via activation of the tyrosine phosphatase SHP2, mitogen-activated protein (MAP) kinase cascade and nuclear factor kappa B (NF-κB) (Brandt et al., 2005), whereas CagL triggers IL-8 induction by activating Src tyrosine kinase, MAP kinase cascade, and NF-κB through direct interaction with the host receptor integrin β_1_ via an arginine-glycine-aspartate (RGD) motif (Gorrell et al., 2013). The *cag* T4SS has also been shown to contribute to IL-6 induction in gastric epithelial cells infected with *H. pylori* (Lu et al., 2005), but the corresponding roles of CagL and CagA remain to be examined.

Although the luminal surface of the gastric epithelium is the primary site of colonization by *H. pylori*, an increasing number of studies suggest that *H. pylori* can gain access to gastric submucosa (Amieva et al., 2003; Semino-Mora et al., 2003; Aspholm et al., 2006; Necchi et al., 2007; Ito et al., 2008). In line with this notion, *H. pylori* has been observed in the vicinity of endothelial cells and even within blood vessels in the gastric submucosa (Aspholm et al., 2006; Necchi et al., 2007). Furthermore, there has been evidence showing that *H. pylori* triggers IL-8 and IL-6 induction upon infection of human endothelial cells (Ding et al., 1997; Innocenti et al., 2002) but the molecular mechanisms involved remained unclear, with no clear correlation observed between *cag*PAI status and the extent of IL-8 or IL-6 induction in primary human umbilical vein endothelial cells (HUVECs) (Innocenti et al., 2002). In this current study we used HUVECs and the human umbilical vein endothelial cell line, EA.Hy926, as host cell models infected with a series of isogenic *H. pylori cag* T4SS mutants to elucidate the molecular mechanisms by which *H. pylori* stimulates IL-8 and IL-6 secretion in human endothelial cells.

## Materials and methods

### Cell culture

AGS cells were maintained in RPMI (Life Technologies) supplemented with 10% (v/v) heat-inactivated fetal bovine serum (FBS) (Life Technologies). The human umbilical vein cell line, EA.Hy926 (ATCC® CRL-2922), were maintained as non-polarized monolayers in Dulbecco's modified Eagle's medium (DMEM) (Life Technologies) supplemented with 4.5 g/L D-glucose, L-glutamine, and 110 mg/L sodium pyruvate (Invitrogen), 10% heat-inactivated FBS, and HAT supplement (Sigma-Aldrich). HUVECs (Catalog number C2519A; Lonza) were maintained as non-polarized monolayers in Endothelial Basal Medium supplemented to Endothelial Growth Medium using the EBM-2™ BulletKit™ (Lonza). Routinely cultured cells and experiments were all maintained at 37°C in a humidified 5% CO_2_ incubator. For experiments where serum-starvation or serum-free conditions were required, cells were grown in culture media without growth factors, additives, or heat-inactivated FBS.

### Bacterial strains and culture conditions

Construction of the various isogenic mutants of *H. pylori* strain P12 has been described in detail previously (Gorrell et al., 2013). *H. pylori* strain 7.13 and its isogenic Δ*cagA* mutant have also been described elsewhere (Franco et al., 2005). *H. pylori* strains were routinely cultured on GC agar (Oxoid) supplemented with 10% (v/v) horse serum (Invitrogen), vitamin mix, vancomycin, and nystatin as described previously (Kwok et al., 2002). For infection experiments, GC agar-cultured *H. pylori* was used to inoculate Heart Infusion (HI) broth (Oxoid) supplemented with 10% (v/v) FBS, 1% (v/v) vitamin mix and 10 μg/ml vancomycin (Sigma-Aldrich). Kanamycin sulfate (15 μg/ml) or chloramphenicol (4 μg/ml) was added as required for the culture of *H. pylori* mutant strains. All *H. pylori* cultures were grown at 37°C under microaerophilic conditions (CampyGen™ system; Oxoid); liquid cultures were incubated with gentle agitation at 120 rpm to an O.D_550nm_ of 0.8–1.2 prior to use in infections.

### Chemical and antibodies

The inhibitors, AG1478 and BMS-345541, were purchased from Merck KGaA. The antibodies used are: PY99 phosphotyrosine-specific mouse monoclonal antibodies (mAb), anti-CagA rabbit polyclonal antibody, and NF-κB p65-specific mouse mAb F-6 (Santa Cruz Biotechnology); anti-EGFR rabbit mAb D38B1 (Cell Signaling); anti- α-tubulin mouse mAb B-5-1-2 (Sigma-Aldrich); goat anti-*H. pylori* antiserum [Kirkegaard and Perry Laboratories (KPL)]; integrin α_v_β_3_-specific mouse mAb LM609 (Millipore); rat mAb AIIB2 (specific for integrin β_1_) (Hall et al., 1990) and BIIG2 (specific for integrin α_5_), developed by C. H. Damsky were obtained as conditioned culture media ultrafiltration concentrates from the Developmental Studies Hybridoma Bank developed under the auspices of the NICHD and maintained by The University of Iowa, Department of Biology, Iowa City, IA 52242. Before use in infection cultures, AIIB2 and BIIG2 concentrates were dialysed against PBS to remove residual antibiotics as described previously (Gorrell et al., 2013).

### Production and heat-inactivation of recombinant CagL

Wild-type or mutant recombinant CagL protein (rCagL), expressed as a fusion protein with a C-terminal hexahistidine-tag, was purified as described previously (Gorrell et al., 2013). Heat-denatured CagL (DN rCagL) was prepared by heating CagL in 50 mM Tris-HCl (pH 8), 100 mM NaCl and 25 mM KCl at 95°C for 30 min.

### Co-culture of cells with *H. pylori* or recombinant CagL

For *H. pylori* infection experiments, all cell-lines were seeded in 24-well plates at 3 × 10^4^ cells per well, and grown for 48 h to achieve ~70% confluence prior to treatment and/or inoculation. For infection experiments, cells were inoculated with either sterile bacterial media alone (HI), or broth-cultured *H. pylori* strains at a multiplicity of infection of 0.1, 1, 10, 30, or 100 colony-forming units per cell, as described for each experiment. For inhibitor studies, cells were washed with PBS and pretreated with small molecule inhibitors or antibodies for 30 min prior to *H. pylori* infection. Spent culture medium was collected at 6 and 24 h-post-infection (hpi) for subsequent analysis by IL-8 or IL-6 ELISA; cell lysates for immunoblot analysis were prepared using Laemmli buffer as described previously (Gorrell et al., 2013). For rCagL treatment, EA-Hy926 monolayers were washed with PBS before adding rCagL^WT^, rCagL^RGA^, or DN rCagL^WT^ diluted in serum-free growth medium; spent culture medium was collected after 24 h incubation.

### Cell attachment assay

The assay was performed as described previously (Kwok et al., 2007) with the following minor modifications. Microtitre plates were coated with PBS alone, or 50 μg/ml of bovine fibronectin (Sigma) or human recombinant vitronectin (Gibco) in PBS at 4°C overnight. Single cell suspensions of HUVECs or AGS were pre-treated with PBS or 2 μg/ml integrin function-blocking antibodies (LM609 or a 1:1 mixture of AIIB2 and BIIG2), for 30 min at 37°C in humidified atmosphere with 5% CO_2._ Treated cells were seeded into the coated microtiter plates at 4 × 10^4^ cell/well, incubated for 2 h, and the wells washed with warm growth media without FBS to remove unbound cells. Attached cells were fixed with 3.8% (w/v) paraformaldehyde (PFA) before visualization using an Olympus CX22 brightfield microscope at 40 × magnification.

### Assessment of cell viability by trypan blue exclusion

To assess the effect of AG1478 on cell viability, HUVEC monolayers (seeded at 6 × 10^4^/well in 24-well plates and incubated for 2 days) were incubated with AG1478 at various concentrations (0.5, 2, 10, or 20 μM) for 24 h. Cells were washed twice with warm growth media, trypsinized and then diluted in 0.4% (w/v) trypan blue in PBS (pH 7.2) for enumeration of viable and non-viable cells using a hemocytometer.

### Field emission scanning electron microscopy (FESEM)

AGS or HUVECs grown on glass coverslips were co-cultured with *H. pylori* strains at MOI of 30 as described above. After 2, 4, and 5 h, samples were washed once with the corresponding growth media and then fixed with 2% PFA at 4°C for 24 h. Fixation was repeated with a fresh batch of 2% PFA at 4°C for 24 h, after which the fixative was replaced with 100 mM HEPES (pH 6.9). Samples were dehydrated and processed by critical point-drying as described previously (Rohde et al., 2003), and were examined with a field emission scanning electron microscope Zeiss DSM 982 Gemini using the Everhart Thornley SE detector and the inlens detectors in a 50:50 ratio. Assessment of the abundance of T4SS pili in each sample was performed blinded. The brightness and contrast of the micrographs were adjusted globally using Adobe Photoshop CS3.

### p65 immunofluorescence

To investigate p65 nuclear translocation during *H. pylori* infection, HUVECs were grown on 12-mm diameter glass coverslips and then serum-starved before *H. pylori*-infection. To examine the effect of the IκB kinase inhibitor BMS345541 on p65 nuclear translocation, HUVECs grown in a 24-well plate were serum-starved and then pre-treated with DMSO or BMS345541 (10 μM) for 30 min prior to co-culture with BHI or *H. pylori* for 3 h. At the required post-infection time points, culture supernatants were recovered and HUVECs monolayers were washed once with PBS before fixation with 4% (w/v) PFA in PBS for 15 min at room temperature. Fixed cells were washed 3 times with PBS, then blocked/permeabilized with PBS (pH 7.2) containing 5% (v/v) FBS and 0.3% (v/v) Triton X-100, before incubation (overnight, 4°C) with anti-p65 mAb and anti-*H. pylori* antisera diluted in antibody diluent (1% (w/v) bovine serum albumin, 0.12% (v/v) Triton X-100, in PBS, pH 7.2). Cells were then washed three times with PBS followed by incubation (2 h at room temperature) with antibody diluent containing AlexaFlour 555-conjugated donkey anti-mouse Ig and AlexaFlour 647-conjugated donkey anti-goat Ig (Invitrogen). Nuclei were counter-stained with DAPI (0.4 μg/ml in PBS; Sigma) for 5 min at room temperature. Localization of p65 and DAPI staining was captured digitally for at least 5 fields per coverslip at 60x magnification using a Leica SP5 confocal microscope. Images were colorized and overlaid using the software ImageJ.

### SDS-polyacrylamide gel electrophoresis (SDS-PAGE) and immunoblot analysis

To examine the relative abundance of total and tyrosyl phosphorylated CagA in *H. pylori*-AGS or *H. pylori*-HUVECs co-cultures, cell lysates were separated in 6% SDS-PAGE gels using the Bio-Rad mini-Protean III system according to the Laemmli buffer system. Tyrosine-phosphorylated and total CagA were detected by immunoblotting using the PY99 and anti-CagA antibodies, respectively, using procedures described previously (Kwok et al., 2007).

To detect EGFR expression in uninfected HUVECs and *H. pylori*-HUVECs co-culture, HUVECs cultures were lysed with radioimmunoprecipitation assay (RIPA) buffer containing 0.5% (w/v) sodium deoxycholate, 150 mM NaCl, 1% (v/v) Tergitol-type NP-40, 50 mM Tris-HCl pH 8.0, 0.1% (w/v) SDS, 10% glycerol, 5 mM EDTA, 20 mM sodium fluoride, 10 μg/mL aprotinin, 1 mM phenylmethane sulfonyl fluoride (PMSF), 10 μg/mL leupeptin, 1 mM sodium orthovanadate, 2.5 mM sodium pyrophosphate and 2.5 mM β-glycerophosphate. Lysates were collected and clarified by high-speed centrifugation. Protein concentrations were then determined with the BCA protein kit (#23225, Thermoscientific) according to the manufacturer's protocol. Protein samples (20–30 μg) were separated in 8% polyacrylamide gels, and were wet transferred onto polyvinylidene difluoride (PVDF) membranes. Membranes were blocked with 5% (w/v) skim milk reconstituted in TBS-T buffer (50 mM Tris-HCl pH 7.5, 150 mM NaCl, 0.1% v/v Tween 20) for 1 h at room temperature. Membranes were then incubated with their respective primary antibody diluted in blocking buffer overnight in 4°C. Membranes were washed thrice for 10 min with TBS-T in room temperature, then probed with horseradish peroxidase-conjugated anti-mouse or anti-rabbit secondary antibody diluted in blocking buffer. Membranes were washed thrice for 10 min with TBS-T in room temperature before signal detection using the enhanced chemiluminescence substrate kit, Western Lightning Plus-ECL (Perkin Elmer).

### IL-8 and IL-6 ELISA

The amounts of IL-8 and IL-6 secreted were determined using the human IL-8 OptEIA ELISA set (BD Biosciences) and human IL-6 OptEIA ELISA set (BD Biosciences), respectively, according to manufacturer's instruction. All samples were measured in duplicate.

### Bacterial adherence assay

HUVECs were seeded in a 96-well plate at a density of 0.5 × 10^4^ cells per well (200 μl) and grown for 2 days before infection with *H. pylori*. At 24 hpi, culture supernatant was discarded and wells were washed twice gently with 300 μl of growth media. Cells were then fixed with 3.8% (w/v) PFA for 15 min at room temperature. After washing with PBS, wells were blocked with 10% FBS in 1 × PBS (200 μl/well) for 1 h at room temperature. After blocking, wells were washed 3 × with PBS supplemented with 0.05% TBST (PBST_0.05_)_._ Cells were then incubated with goat anti-*H. pylori* antiserum (100 μl/well) diluted 1:1,000 in blocking buffer for 1 h at room temperature. After washing with PBS three times, rabbit anti-goat IgG horseradish peroxidase-conjugated antibody (100 μl/well) diluted 1:2,000 in blocking buffer was added and incubated for 1 h at room temperature. After washing five times with PBST_0.05_, cells were incubated with 3,3′,5,5′-Tetramethylbenzidine (TMB) (100 μl/well) for 25–45 min, after which the reaction was stopped by addition of 1M H_2_SO_4_ (50 μl/well). Optical density (O.D.) at 450 nm of each well was measured using a TECAN ELISA plate reader and background-corrected against absorbance at 570 nm.

### Determination of EGR1 and EGFR mRNA levels by real-time PCR

HUVECs were seeded at 3 × 10^4^ cells per well into 24-well plates and grown to ~70% confluence (2.5 days). Cells were serum-starved for 5 h before inoculation with sterile BHI, or *H. pylori* P12 WT or isogenic *cagL* or *cag*PAI deletion mutant strains (MOI = 50). Cells were harvested at 0.5, 1, 2, and 3 hpi into 350 μl RLY cell lysis buffer (ISOLATE II RNA mini kit, Bioline) supplemented with 10 mM dithiothreitol, and RNA purified according to the kit protocol for “purifying total RNA from cultured cells and tissue.” RNA concentrations were estimated by Nanodrop (Thermo Fisher) and 125 ng RNA was used for cDNA production using the Superscript III first strand synthesis system with oligo d(T) primers (Thermo Fisher) according to the kit protocol. *EGR1* and *EGFR* mRNA levels were assessed by qPCR using FastStart Universal SYBR Green Master mix (Roche) with gene-specific primers for *EGR1* [EGR1F 5′-CCCGTTCGGATCCTTTCCT-3′ and EGR1R 5′-CAGCATCATCTCCTCCAGCTT-3′ (Keates et al., 2005)], *EGFR* [EGFR-F 5′-GCGTCTCTTGCCGGAATGT-3′ and EGFR-R 5′-GGCTCACCCTCCAGAAGGTT-3′ (Keates et al., 2007)] and *GAPDH* [GAPDH-F 5′ CGGGAATGCAGTTGAGGATC 3′ and GAPDH-R 5′ AGGATGGTGTAAGCGATGGC 3′ (Witta et al., 2009)]. Quantitative PCRs were prepared in triplicate and individual 25 μl reactions contained 0.4 mM primer pair and 2 μl cDNA (≈12.5 ng RNA) or nuclease-free H_2_O (no-template control). Cycling conditions were: 1 cycle (95°C for 10 min); 40 cycles (95°C for 15 s, 60°C for 60 s); melting curve from 65 to 95°C, 5 s per 1°C. Output qPCR data was assembled into amplicon groups and analyzed using LinRegPCR [software version 2017.0 (Ramakers et al., 2003)]; mean PCR efficiencies were 2.01 for *GAPDH*, 2.03 for *EGR1* and 2.06 for *EGFR*. Fold differences in *EGR1* and *EGFR* mRNA levels calculated using GAPDH as the reference gene and BHI as the control sample by the ΔΔCT (2∧-((Ct_target gene_ – Ct_ref gene_)_control sample_ – (Ct_target gene_ – Ct_ref gene_)_test sample_)) and the Ruijter ((PCR efficiency^Ct(ref gene)^ / PCR efficiency^Ct(target gene)^)_test sample_ / (PCR efficiency^Ct(ref gene)^ / PCR efficiency^Ct(target gene)^)_control sample_) methods (Ruijter et al., 2009) were indistinguishable; differential expression was reported as 2^−ΔΔCT^ values.

### Statistical analyses

Numerical data were analyzed using Prism 6.0 (GraphPad software) and presented as mean ± standard error of the mean (SEM) or mean ± standard deviation (*SD*). N denotes the number of independent experiments performed. Statistical significance was determined using Student's *t*-test, one-way ANOVA or two-way ANOVA with appropriate post-test, as indicated in the figure legends.

## Results

### *H. pylori* is a potent stimulant of proinflammatory responses by endothelial cells

**Figure 1:**
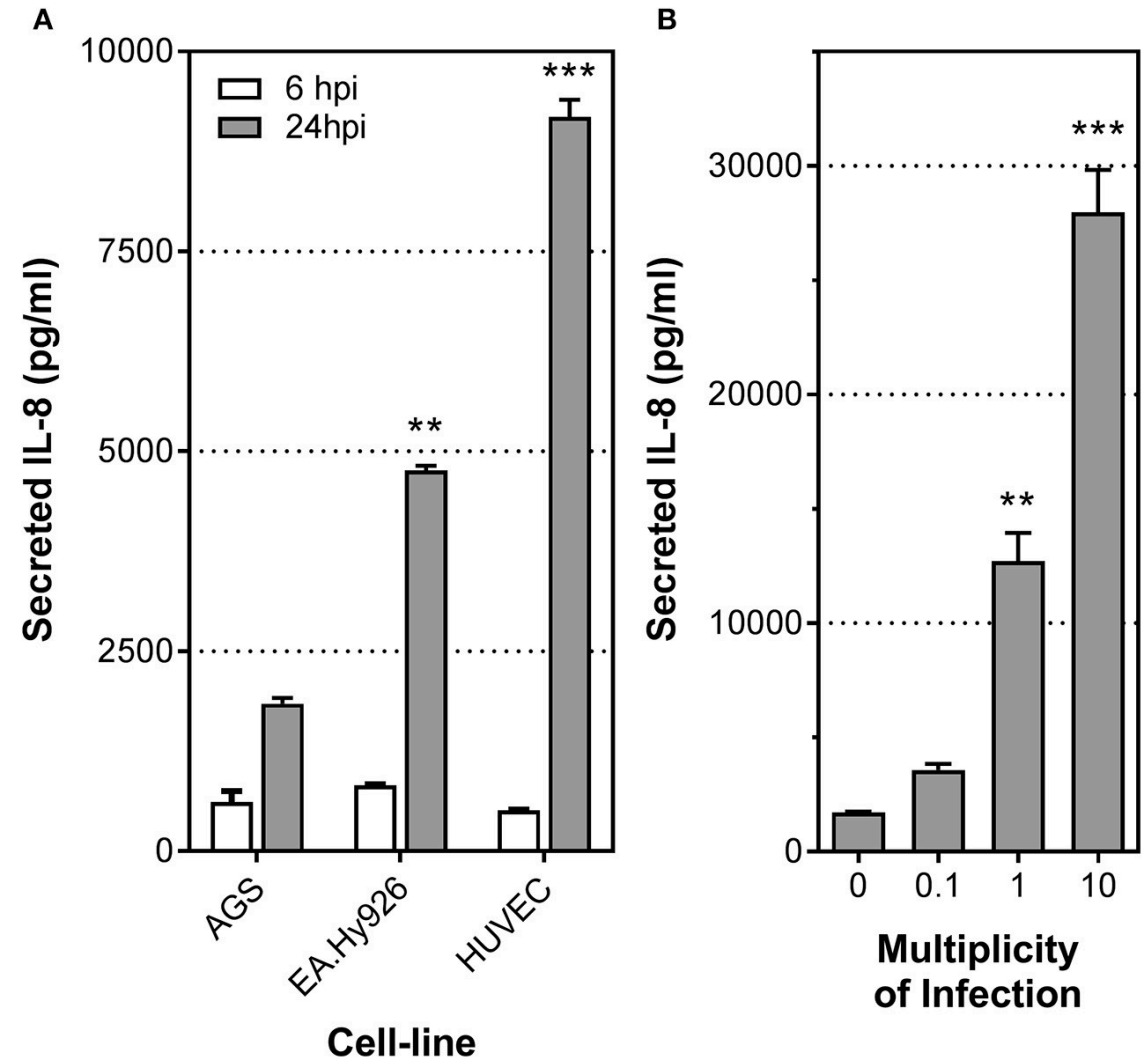
*H. pylori* stimulates potent inflammatory response in human endothelial cells. **(A)** Mean secreted IL-8 levels in spent culture media collected at 6 and 24 h post-infection (hpi) from AGS, EA.Hy925 or HUVECs infected with *H. pylori* P12 wild-type at MOI = 1. Error bars denote SEM; *N* = 2; statistical analysis using one-way ANOVA (Tukey's multiple comparisons post-test); ^**^ and ^***^ denote conditions significantly different (*p* < 0.005 and *p* < 0.001, respectively) to AGS. **(B)** Mean secreted IL-8 levels in spent culture media collected at 24 hpi from HUVECs inoculated with HI, or with *H. pylori* P12 wild-type at MOI = 0.1, 1, or 10. Error bars denote SEM; *N* = 2; statistical analysis using one-way ANOVA (Tukey's multiple comparisons post-test); ^**^ and ^***^ denote conditions significantly different (*p* < 0.005 and *p* < 0.001, respectively) to MOI of 0.

Previous studies have shown that *H. pylori* stimulates secretion of the proinflammatory cytokines IL-8 and IL-6 upon infection of endothelial cells (Ding et al., 1997; Innocenti et al., 2002). To further understand the biological significance of these responses and the mechanism behind, we first compared the levels of IL-8 secretion induced by *H. pylori* upon infection of primary HUVECs, an immortalized HUVEC line (EA.Hy926), or a gastric epithelial cell line (AGS) (Figure 1A). Low multiplicities of infection (MOI) were used to mimic the relatively low abundance of *H. pylori* present in the gastric submucosa where the majority of blood vessels reside. At MOI of 1, similar levels of IL-8 induction were observed at 6 h post-inoculation in all three cell lines/types upon infection with the *H. pylori* strain P12. However, after 24 h of stimulation by *H. pylori*, the level of IL-8 secreted by the endothelial cell line EA.Hy926 was more than double that observed with AGS (Figure 1A). This marked response was even more pronounced with the primary endothelial cells HUVECs (Figure 1A). Moreover, these levels of IL-8 induction observed in *H. pylori*-infected endothelial cells at MOI of 1 are similar to or even higher than that previously observed with *H. pylori* infection of primary human gastric epithelial cells at MOI of 100 (Fischer, 2011; Mustapha et al., 2014). In fact, *H. pylori* induced IL-8 secretion by primary endothelial cells even at MOI of 0.1 (Figure 1B). Taken together, these results suggest that endothelial cells may play a more important role in mounting early proinflammatory responses toward *H. pylori* than previously envisaged.

### The T4SS is essential for *H. pylori*-induced proinflammatory responses in endothelial cells

**Figure 2:**
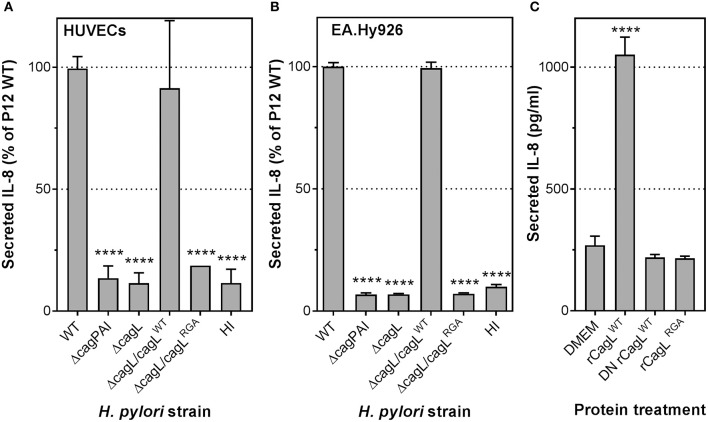
*H. pylori* T4SS, via the CagL adhesin, plays an essential role in IL-8 induction in infected human endothelial cells. Mean secreted IL-8 levels in spent culture medium collected from **(A)** HUVECs or **(B)** EA.Hy926 cells inoculated with heart infusion broth (HI), *H. pylori* P12 wild-type (WT), or isogenic Δ*cagPAI* and Δ*cagL* mutants at 24 hpi; MOI = 1. Error bars denote SEM, *N* = 3; statistical analysis using two-way ANOVA (Tukey's multiple comparisons post-test); ^****^denotes conditions significantly different (*p* < 0.0001) to WT. **(C)** Mean secreted IL-8 levels in spent culture medium collected from EA.Hy926 cells incubated for 24 h with DMEM media or 100 μg/ml recombinant CagL wild-type (rCagL^WT^), heat-inactivated CagL (DN rCagL^WT^) or CagL RGA mutant protein (rCagL^RGA^). Error bars denote *SD, N* = 4; statistical analysis using one-way ANOVA (Tukey's multiple comparisons post-test).

To elucidate the molecular mechanism(s) involved in stimulating the potent inflammatory response in endothelial cells by *H. pylori*, we examined the contribution of *H. pylori* T4SS to IL-8 induction. HUVECs were infected with *H. pylori* P12 wild-type (WT) or isogenic *cag*PAI-deletion (Δ*cag*PAI) mutant. The Δ*cag*PAI mutant, which lacks the genes that encode the T4SS, lost the ability to induce IL-8 secretion in HUVECs compared to wild-type *H. pylori* (Figure 2A). Similar observations were obtained upon *H. pylori* infection of the endothelial cell line EA.Hy926 (Figure 2B). These findings suggest that induction of IL-8 by *H. pylori* is strongly dependent upon the constituents of the T4SS.

### CagL is an essential trigger of proinflammatory responses in endothelial cells

CagL is a T4SS-associated adhesin capable of binding to the human transmembrane receptor integrin α_5_β_1_ via an arginine-glycine-aspartate (RGD) motif (Kwok et al., 2007). We have shown previously that interaction of CagL with integrin β_1_ via the RGD motif triggers secretion of IL-8 in gastric epithelial cells (Gorrell et al., 2013). To test whether CagL and its RGD motif are also important for stimulating IL-8 secretion upon *H. pylori* infection of endothelial cells, we infected HUVECs with a *H. pylori* P12 isogenic mutant deficient in CagL (P12Δ*cagL*) or an isogenic *cagL* mutant that expresses an arginine-glycine-alanine (RGA) motif instead of the RGD motif (P12Δ*cagL*/*cagL*^RGA^). The Δ*cagL* mutant, like the Δ*cag*PAI mutant, was significantly attenuated in its ability to induce IL-8 secretion in HUVECs (Figure 2A), as well as the EA.Hy926 cell-line (Figure 2B). Similarly, substitution of the RGD motif with RGA in CagL (Δ*cagL*/*cagL*^RGA^) resulted in severely attenuated IL-8 induction (Figure 2A). In contrast, knock-in of P12Δ*cagL* with wild-type *cagL* (P12*cagL*^WT^) fully restored IL-8 secretion, confirming that the loss of P12Δ*cagL*'s ability to induce IL-8 was not due to polar effects (Figures 2A,B). Adherence assays confirmed that the mutants and wild-type strains adhered to endothelial host cells to similar extents (Supplementary Figure 1). Taken together, these results suggest that CagL and its RGD motif play a major role in inducing IL-8 secretion upon *H. pylori* infection of HUVECs, which is reminiscent of the key role of CagL in IL-8 induction in infected gastric epithelial cells (Gorrell et al., 2013).

We have previously shown that CagL protein alone was sufficient to induce IL-8 secretion upon *H. pylori* infection of gastric epithelial cells (Gorrell et al., 2013). To test whether the same holds true for *H. pylori*-mediated IL-8 induction in endothelial cells, EA.Hy926 cells were stimulated with recombinant wild-type CagL protein (rCagL^WT^), heat-denatured recombinant CagL (DN rCagL^WT^) or CagL^RGA^ mutant protein. The results showed that rCagL^WT^, but not DN rCagL^WT^ or the rCagL^RGA^ mutant, stimulated an ~3.5-fold increase in IL-8 secretion by endothelial cells (Figure 2C); the inability of DN rCagL^WT^ to induce an IL-8 response confirmed that the response was unlikely to be caused by any trace amount of contaminating lipopolysaccharide (LPS) as the pyrogenic effect of LPS is heat-resistant (Gao et al., 2006). These observations indicate that CagL alone is sufficient to trigger IL-8 secretion by endothelial cells in an RGD-dependent manner.

### CagL triggers IL-8 and IL-6 secretion by endothelial cells in a manner independent of CagA translocation

**Figure 3:**
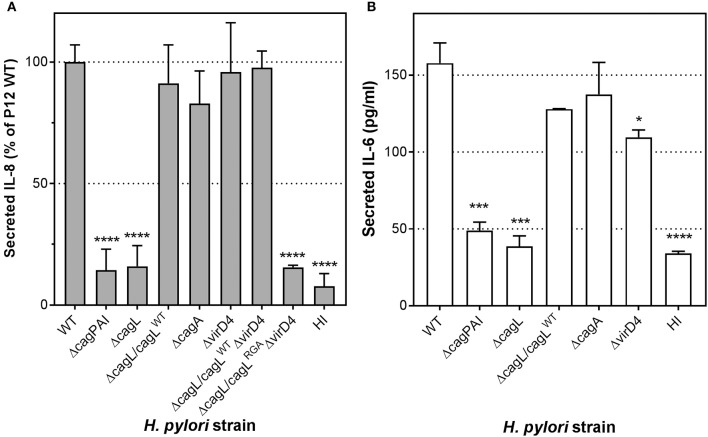
The essential role CagL plays in IL-8 and IL-6 induction in infected human endothelial cells is independent of CagA. **(A)** Mean secreted IL-8 levels in spent culture medium collected at 24 hpi from HUVECs inoculated with HI, or *H. pylori* strains P12 WT or various isogenic Δ*cagL*, Δ*cagA*, and Δ*virD4* single and double mutants at MOI = 10. Error bars denote SEM, *N* = 4; statistical analysis using two-way ANOVA (Tukey's multiple comparisons post-test); ^****^denotes conditions significantly different (*p* < 0.0001) to WT. **(B)** Mean secreted IL-6 levels in spent culture medium collected at 24 hpi from HUVECs inoculated with HI, or *H. pylori* strains P12 WT or various isogenic Δ*cagL*, Δ*cagA*, and Δ*virD4* mutants at MOI = 10. Error bars denote *SD, N* = 2; statistical analysis using two-way ANOVA (Tukey's multiple comparisons post-test); ^*^, ^***^, and ^****^ denote conditions significantly different (*p* < 0.05, *p* < 0.001, and *p* < 0.0001, respectively) to WT.

During *H. pylori* infection of gastric epithelial cells, CagL plays a major role in mediating the translocation of the T4SS substrate, CagA, into the host cells (Kwok et al., 2007; Gorrell et al., 2013). Moreover, translocated CagA can contribute to IL-8 induction in *H. pylori*-infected gastric epithelial cells (Brandt et al., 2005). It is thus possible that the CagL-dependent IL-8 induction observed in endothelial cells could be mediated by translocated CagA *per se*. To investigate whether CagA or CagA translocation contributes to endothelial cell proinflammatory responses, we infected HUVECs with wild-type *H. pylori, cagA*-deletion (Δ*cagA*) or *virD4*-deletion (Δ*virD4*) mutant. The Δ*virD4* mutant is defective in CagA translocation as VirD4 is the coupling protein of the *H. pylori* T4SS essential for the translocation of CagA through the T4SS channel into the host cell (Fischer, 2011). In contrast to the Δ*cag*PAI and Δ*cagL* mutants, the Δ*cagA* or Δ*virD4* mutants were fully capable of triggering IL-8 secretion in HUVECs to levels observed with wild-type (Figure 3A). Adherence assays confirmed that these mutants and wild-type strains adhered to HUVECs to similar extents (Supplementary Figure 1). These observations therefore show that the *H. pylori* T4SS *per se*, but not its translocation substrate CagA, is a major inducer of IL-8 secretion in *H. pylori*-infected endothelial cells. Moreover, in agreement with the IL-8 results, analysis of IL-6 secretion in samples from this series of experiments indicated that *H. pylori*-mediated induction of IL-6 in infected HUVECs was also T4SS- and CagL-dependent, but CagA-independent (Figure 3B).

Given that the IL-8 induction by *H. pylori* in HUVECs was significantly more pronounced compared to the IL-6 response, we decided to focus on the IL-8 response in our further interrogation of the molecular mechanism by which the *H. pylori* T4SS triggers proinflammatory responses in endothelial cells. To further test the hypothesis that CagL can directly stimulate IL-8 induction in gastric epithelial cells in a manner independent of its role in mediating CagA translocation (Gorrell et al., 2013), we made use of the *cagL*^WT^Δ*virD4* mutant and the *cagL*^RGA^Δ*virD4* double mutant. The *H. pylori cagL*^WT^Δ*virD4* mutant, which expresses wild-type CagL but lacks VirD4, is defective in CagA translocation but still able to induce IL-8 secretion in gastric epithelial cells; in contrast, the *cagL*^RGA^Δ*virD4* double mutant is defective in both CagA translocation and IL-8 induction in infected gastric epithelial cells (Gorrell et al., 2013). Upon infection of HUVECs, the *cagL*^WT^Δ*virD4* variant was fully capable of potent IL-8 induction, but the *cagL*^RGA^Δ*virD4* mutant was significantly attenuated in its ability to induce IL-8 (Figure 3A). These results support the notion that CagL and its RGD motif contribute to IL-8 induction in endothelial cells in a manner independent of their roles in CagA translocation.

We next examined whether the CagA-independent effect is unique to the *H. pylori* strain P12. HUVECs were stimulated with wild-type *H. pylori* strain 7.13 and its isogenic Δ*cagA* mutant. Strain 7.13 is a carcinogenic Mongolian gerbil-adapted *H. pylori* human isolate which possesses a functional T4SS and is able to translocate a relatively high abundance of CagA into the gastric epithelial cell line AGS compared to other strains (Franco et al., 2005). Consistent with the hypothesis that CagA is dispensable for IL-8 induction by *H. pylori* upon infection of human endothelial cells, no difference in the levels of IL-8 induction was observed between wild-type 7.13 and 7.13Δ*cagA* during infection of HUVECs (Supplementary Figure 2).

Taken together, these novel findings support the notion that the *H. pylori* T4SS and its adhesin, CagL, are key drivers of IL-8 induction during *H. pylori* infection of endothelial cells. Moreover, this pro-inflammatory response occurs in a manner independent of CagA translocation.

### Interaction of *H. pylori* with HUVECs does not result in CagA phosphorylation or formation of *cag*PAI-dependent pili

**Figure 4:**
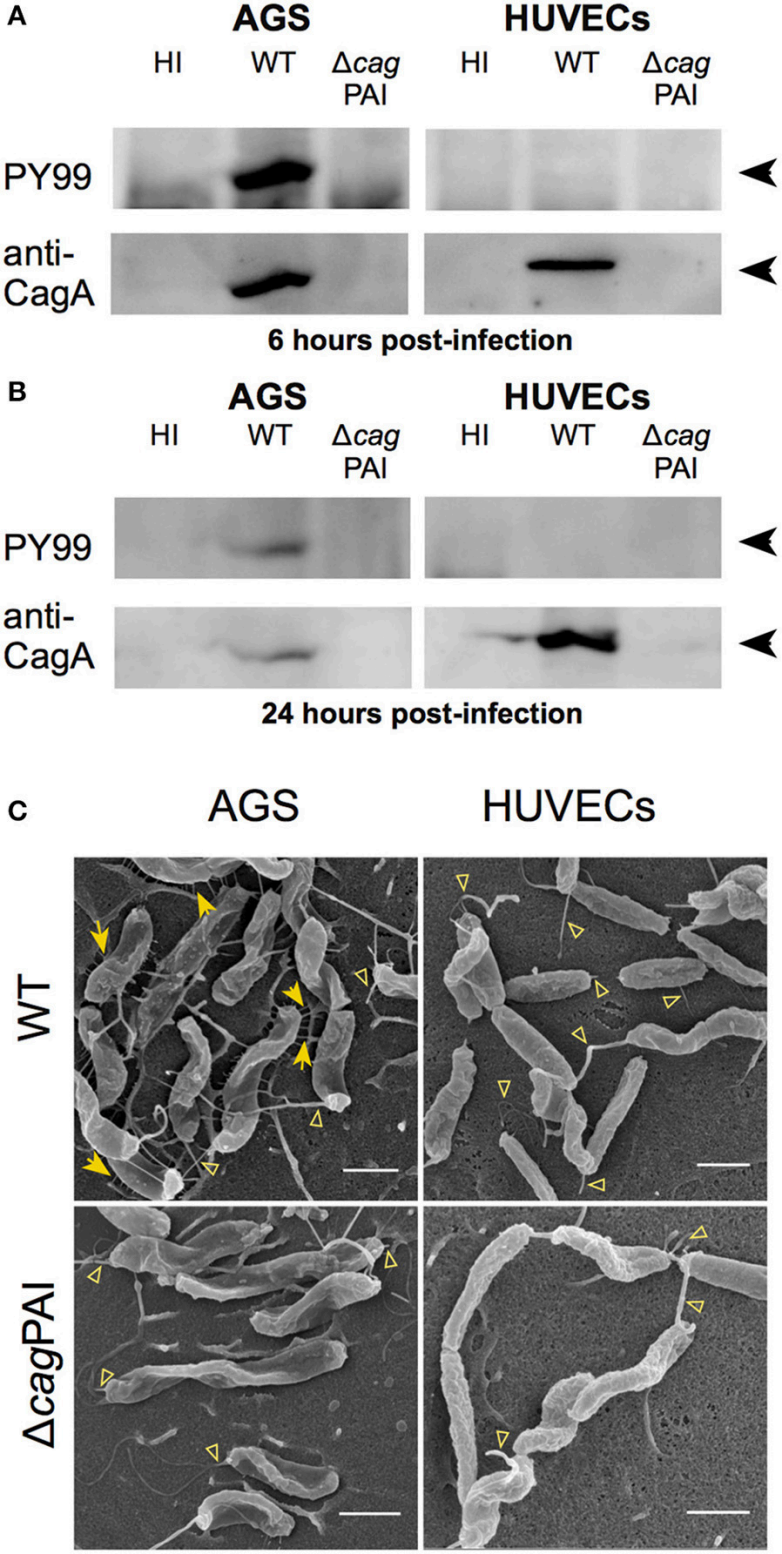
Interaction of *H. pylori* with HUVECs does not result in CagA translocation or formation of *cag*PAI-dependent pili. Analysis of CagA translocation upon **(A)** 6 hpi or **(B)** 24 hpi infection of AGS or HUVECs with *H. pylori* P12 WT or isogenic Δ*cagPAI* mutant; MOI = 30. Infected-cell lysates were analyzed by western blot using antibody PY99 to detect tyrosyl-phosphorylated CagA. Blots were stripped and re-probed with CagA-specific antibody to determine the total amounts of CagA. Black arrowheads indicate CagA or tyrosine phosphorylated CagA bands. **(C)** AGS or HUVECs were infected with *H. pylori* WT or Δ*cagPAI* mutant at MOI of 30. Samples were fixed at 4 hpi and then subjected to FESEM. Yellow arrows indicate *cag*PAI-dependent pili-like structures; open arrowheads indicate flagella, including intact flagella and remnants of broken sheathed or unsheathed flagella. Scale bars, 1 μm.

The observation that CagA does not play a role in IL-8 induction in *H. pylori*-infected endothelial cells led us to speculate that the *H. pylori* T4SS may lack the ability to translocate CagA into these cells. Previous studies have shown that CagA, upon translocation by *H. pylori* T4SS into gastric epithelial cells, becomes phosphorylated by the host Src kinase on a number of tyrosine residues in its C-terminal region (Segal et al., 1999; Backert et al., 2000; Stein et al., 2000), which is required for CagA to trigger IL-8 secretion in *H. pylori*-infected gastric epithelial cells (Brandt et al., 2005). Therefore, we investigated whether *H. pylori* infection of HUVECs results in tyrosyl-phosphorylated CagA. No tyrosyl-phosphorylated CagA could be detected in the lysate of *H. pylori*-infected HUVECs up to 24 hpi, whereas the presence of tyrosyl-phosphorylated CagA was readily detectable in *H. pylori*-infected AGS cells (Figures 4A,B, and Supplementary Figure 3), indicating that *H. pylori* is able to efficiently translocate CagA into AGS, whereas in HUVECs CagA was either not translocated or not tyrosyl-phosphorylated. Supporting the proposition that CagA translocation occurs in infected AGS but not infected HUVECs is the observation that *cag*PAI-dependent pili-like structures were abundantly present at the interface of *H. pylori* and AGS (Figure 4C, left-hand panels) whereas no pili-like structures were detected at the interface of *H. pylori* and HUVECs (Figure 4C, right-hand panels). The elaboration of *cag*PAI-dependent pili-like structures at the interface of *H. pylori* and AGS cells has been associated with active CagA translocation into the host cell (Rohde et al., 2003; Kwok et al., 2007; Shaffer et al., 2011). Thus, taken together, these observations suggest that although the T4SS is capable of triggering proinflammatory responses in *H. pylori*-infected HUVECs, it does not translocate CagA into or lead to CagA phosphorylation in HUVECs.

### Integrins α_5_β_1_ and α_v_β_3_ are dispensable for *H. pylori*-induced IL-8 secretion by endothelial cells

**Figure 5:**
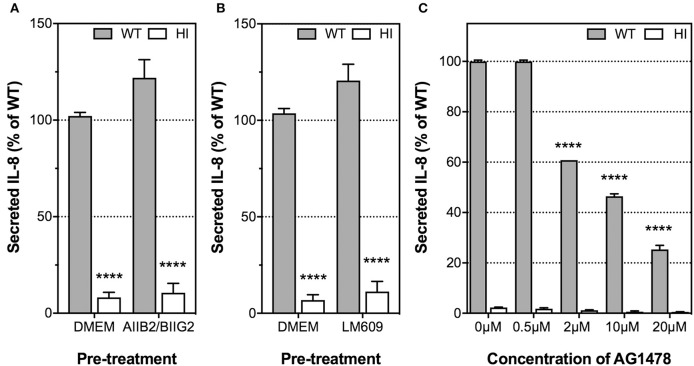
The roles of integrin α_5_β_1_, integrin α_v_β_3_, and EGFR in IL-8 induction by *H. pylori* upon infection of HUVECs. Prior to incubation with *H. pylori* P12 (MOI =1) or HI broth, HUVECs were pre-treated with the function-blocking antibodies **(A)** AIIB2 and BIIG2 (2 μg/ml each), **(B)** LM609 (2 μg/ml), or **(C)** EGFR small molecule inhibitor AG1478 at various concentrations indicated. Spent culture media was harvested at 24 hpi and assayed by ELISA for secreted IL-8. IL-8 levels are expressed as the mean percentage of that determined for P12 WT-infected HUVECs. **(A,B)** Error bars denote SEM, *N* = 3; statistical analysis using two-way ANOVA (Tukey's multiple comparisons post-test); comparisons between infected and uninfected shown (^****^p < 0.0001); antibody-treated and untreated samples were not significantly different. **(C)** Error bars denote SEM, *N* = 2. Statistical analysis of AG1478 dose-response by two-way ANOVA (Tukey's multiple comparisons post-test); significant differences compared to 0 μM are shown; ^****^p < 0.0001.

Having ascertained that the *H. pylori* T4SS and, in particular, CagL, played an important role in IL-8 induction, we next examined whether the proinflammatory response is mediated by the host receptor integrin α_5_β_1_, as previously observed for IL-8 induction by *H. pylori* in AGS cells (Hutton et al., 2010; Gorrell et al., 2013). The role of RGD-binding integrin α_v_β_3_ in *H. pylori*-mediated IL-8 secretion by endothelial cell was also investigated as integrin α_v_β_3_ is abundantly expressed in HUVECs (Brooks et al., 1994; Baranska et al., 2005) and is crucial for endothelial functions such as angiogenesis and adhesion of endothelial cells to vitronectin in the extracellular matrix (Alghisi et al., 2009). To this end, we used the β_1_ integrin function-blocking antibody, AIIB2, together with the α_5_ integrin-function blocking antibody, BIIG2, to block α_5_β_1_ function; and the α_v_β_3_ integrin-function blocking antibody, LM609. The specific inhibitory activities of AIIB2/BIIG2 and LM609 for cell spreading on their individual ligands fibronectin or vitronectin, respectively, was first confirmed in cell attachment assays (Supplementary Figure 4). HUVECs were then pre-treated with these antibodies prior to *H. pylori* infection and detection of IL-8 secretion. Integrin α_5_β_1_ function was not essential for IL-8 induction by *H. pylori* in HUVECs, as pre-treatment of HUVECs with AIIB2 and BIIG2 did not suppress IL-8 secretion in response to *H. pylori* (Figure 5A). Similar observations were obtained when HUVECs were pre-treated with LM609 prior to *H. pylori* infection (Figure 5B). These findings indicate that integrins α_5_β_1_ and α_v_β_3_ are dispensable for *H. pylori*-mediated IL-8 induction upon infection of endothelial cells.

### Epidermal growth factor receptor (EGFR) plays an important role in *H. pylori*-induced IL-8 and IL-6 secretion by endothelial cells

Previous studies have shown that *H. pylori* is capable of transactivating epidermal growth factor receptor (EGFR) and up-regulating its expression in gastric epithelial cells in a T4SS-dependent manner, and that EGFR activation contributes to IL-8 induction by *H. pylori* (Keates et al., 2001, 2005, 2007). To investigate whether EGFR also plays a role in the induction of IL-8 by *H. pylori* during infection of HUVECs, we pre-treated HUVECs with AG1478 prior to infection with *H. pylori*. AG1478 is a small-molecule inhibitor that specifically inhibits the tyrosine kinase activity of EGFR (Zhu et al., 2001). AG1478 strongly inhibited IL-8 secretion (Figure 5C) and IL-6 secretion (Supplementary Figure 5) by HUVECs in response to *H. pylori* in a dose-dependent manner without affecting cell viability at concentrations up to 20 μM (Supplementary Figure 6). These findings indicate that EGFR function is a major contributor to IL-8 induction by *H. pylori* in HUVECs.

**Figure 6:**
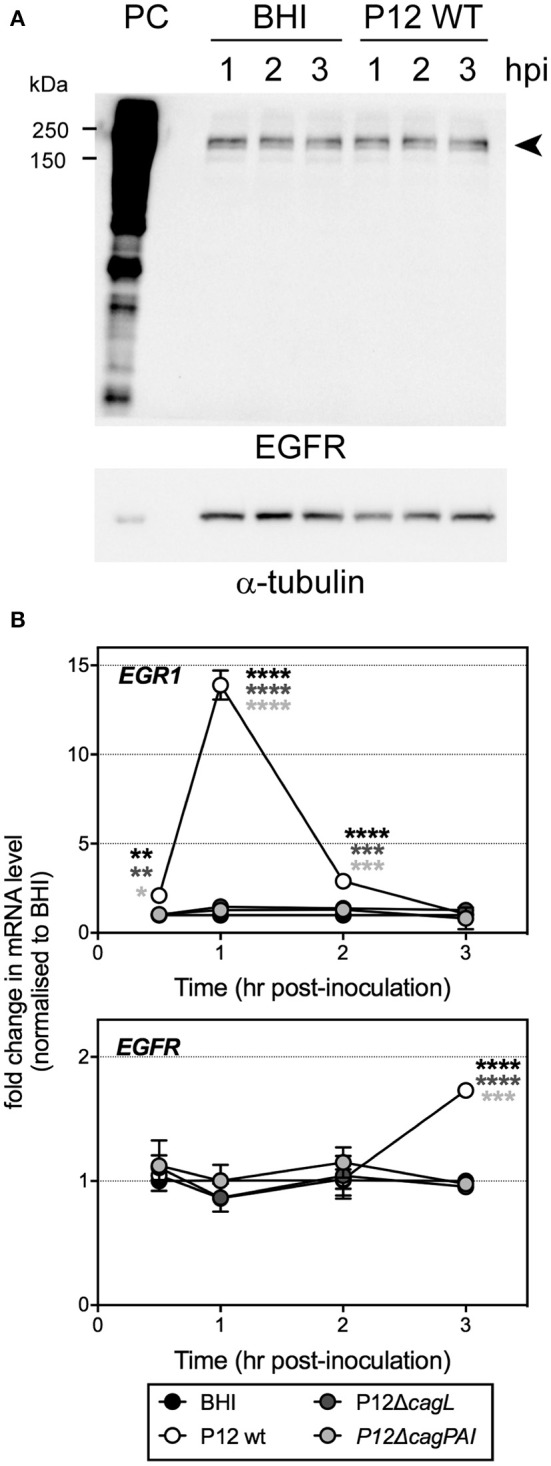
EGFR expression in HUVECs and upregulation of EGR1 mRNA level upon infection of HUVECs with *H. pylori* P12. **(A)** HUVECs were inoculated with BHI broth or *H. pylori* P12 WT (MOI = 50). Cell lysates (30 μg) were harvested at 1, 2, and 3 h post-inoculation for quantitation of EGFR protein level by Western blot. Whole cell lysate (20 μg) of the basal MDA-MB 468 triple negative breast cancer cell line that abundantly expresses EGFR was used as a positive control (PC) for EGFR expression and detection. α-tubulin was used as loading control. Arrowhead indicates EGFR bands. In line with previous reports, two EGFR bands were detected, which most likely correspond to EGFR isoforms that differ in post-translational modification (Gabius et al., 2012; He et al., 2016). **(B)** HUVECs were inoculated with BHI broth, or *H. pylori* P12 WT or isogenic Δ*cagL* and Δ*cag*PAI mutant strains (MOI = 50), and cells were harvested at 0.5, 1, 2, and 3 hpi for quantitation of *EGR1* (upper panel) and *EGFR* (lower panel) mRNA levels by qRT-PCR. *EGR1* mRNA levels in response to *H. pylori* P12 WT was significantly greater than BHI, P12Δ*cagL*, and P12Δ*cag*PAI (asterisk shades match corresponding graph symbols) at 0.5, 1, and 2 hpi. *EGFR* mRNA levels in response to *H. pylori* P12 WT was significantly greater than BHI, P12Δ*cagL* and P12Δ*cag*PAI (asterisk shades match corresponding graph symbols) at 3 hpi. Statistical significance was determined by two-way ANOVA with Tukey's multiple comparison post-test; ^*^P < 0.05, ^**^P < 0.01, ^***^P < 0.001, ^****^P < 0.0001; all other comparisons not significantly different. Mean ± *SD*, data combined from two independent HUVEC activation experiments.

Due to the rather low level of EGFR expression in HUVECs (Figure 6A), we were unable to detect activation of EGFR directly by Western blot using antibodies specific for the various tyrosine phosphorylated forms of EGFR, or by immunoprecipitating the EGFR and then blotting with such antibodies or with anti-phosphotyrosine antibodies. However, it is known that transactivation of EGFR by *H. pylori* upregulates expression of the transcription factor early growth response gene 1 (EGR1) in the gastric epithelial cell line AGS (Keates et al., 2005). The role of EGR1 as a downstream effector of EGFR is well established. We therefore used EGR1 upregulation as a marker for EGFR activation and examined whether T4SS or CagL is involved in the activation of EGFR during infection. First, our results showed that *H. pylori* P12 WT was able to efficiently upregulate EGR1 expression in HUVECs. EGR1 upregulation was detectable as early as after 0.5 hpi and was maximal at 1 hpi (14-fold of upregulation compared to uninfected), with the EGR1 expression level returning to baseline by 3 hpi (Figure 6B, upper panel). The response followed a pattern very similar to that observed upon stimulation of gastric epithelial cells by *H. pylori* (Keates et al., 2005). Moreover, EGR1 upregulation was dependent on T4SS and CagL as both the Δ*cag*PAI and Δ*cagL* mutants, in contrast to wild type, were unable to upregulate EGR1 expression (Figure 6B, upper panel). These findings therefore suggest that *H. pylori* T4SS and the T4SS adhesin CagL play essential role(s) in activating EGFR during infection of HUVECs. In addition to stimulating EGFR activity, *H. pylori* P12 WT also triggered a moderate upregulation of EGFR expression in HUVECs as shown by a 1.7-fold increase in EGFR mRNA level at 3 hpi compared to uninfected control (Figure 6B, lower panel). In contrast, the Δ*cag*PAI and Δ*cagL* mutants had no effect on EGFR expression (Figure 6B, lower panel), suggesting that upregulation of EGFR expression by *H. pylori* in HUVECs depends on T4SS and CagL. Taken together, these findings indicate that *H. pylori* triggers both activation and upregulation of expression of EGFR in HUVECs in a T4SS- and CagL-dependent manner.

### Nuclear factor kappa B (NF-κB) is activated by *H. pylori* T4SS and plays a key role in IL-8 induction in *H. pylori*-infected endothelial cells

**Figure 7:**
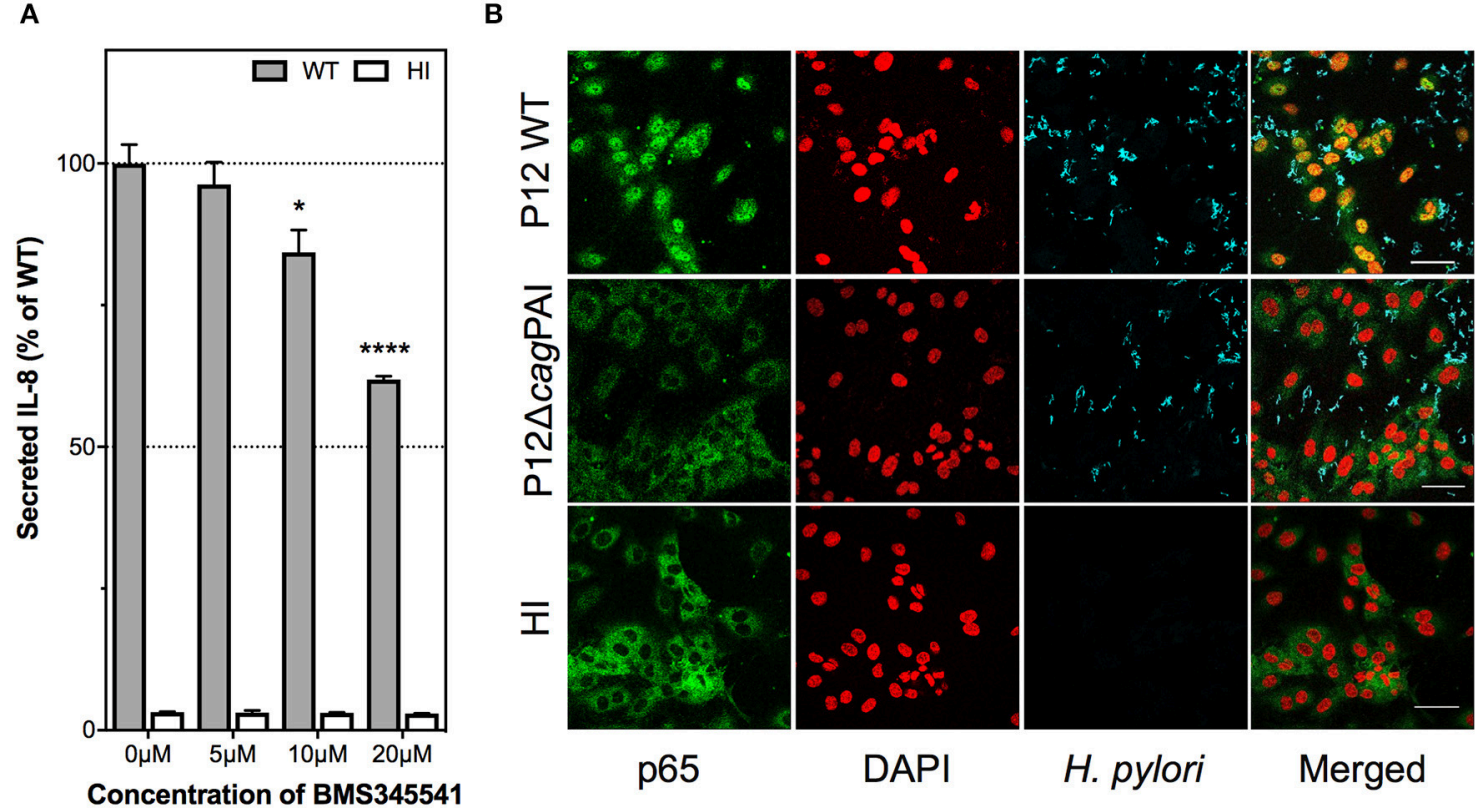
Contribution of NF-κB activation to *H. pylori*–induced IL-8 secretion by human endothelial cells. **(A)** IL-8 induction is dependent upon NF-κB activation. HUVECs were pre-treated with BMS345541 at 5, 10, or 20 μM prior to inoculation with HI or *H. pylori* P12. Spent culture media was harvested at 24 hpi and assayed by ELISA for secreted IL-8; IL-8 levels are expressed as the mean percentage of that determined with P12 WT-infected HUVECs. Error bars denote SEM, *N* = 2; statistical analysis of inhibitor dose-response by two-way ANOVA (Tukey's multiple comparisons post-test); significant differences compared to 0 μM are shown; ^*^p < 0.05; ^****^p < 0.0001. **(B)** *H. pylori* activates nuclear translocation of NF-κB subunit p65 upon infection of HUVECs. HUVECs inoculated with HI broth, or *H. pylori* strains P12 WT or P12 *cag*PAI mutant (MOI = 30) were fixed at 3 hpi, and immunolabeled for NF-κB p65 (green) and *H. pylori* (blue); nuclei were counterstained with DAPI (red); yellow/orange in merged images denotes nuclear p65. Scale bar, 50 μm. Images shown are representative of two independent experiments.

IL-8 induction during *H. pylori* infection of gastric epithelial cells has been shown to require NF-κB activation (Keates et al., 2007; Gorrell et al., 2013). To test the hypothesis that IL-8 induction by *H. pylori* in endothelial cells is also mediated via activation of NF-κB, HUVECs were pre-treated with the highly specific IκB kinase inhibitor, BMS-345541, which specifically inhibits NF-κB activation (Burke et al., 2003), prior to *H. pylori* infection. BMS-345541 significantly inhibited IL-8 induction by *H. pylori* in HUVECs in a dose-dependent manner (Figure 7A). Trypan blue exclusion assays confirmed that the inhibitor concentrations used had no detectable effect on the viability of HUVECs (data not shown). Additionally, immunofluorescence analysis showed that *H. pylori* stimulated activation of NF-κB, which was measured as a function of nuclear translocation of the NF-κB p65 subunit, in a *cag*PAI-dependent manner at 3 hpi of HUVECs (Figure 7B) with the effect being observable up to 7 hpi (data not shown). BMS-345541 at a concentration of 10 μM significantly blocked p65 nuclear translocation in *H. pylori*-infected HUVECs (Supplementary Figure 7). These findings corroboratively indicate that *H. pylori* T4SS triggers NF-κB activation and that activation of NF-κB contributes to IL-8 induction in *H. pylori*-infected endothelial cells.

## Discussion

During chronic infection, the pronounced host immune responses triggered by *H. pylori* infection cause damage to the gastric epithelial layer (Kusters et al., 2006). Such tissue damage reduces epithelial integrity, allowing *H. pylori* to gain access to other cell types in the gastric mucosa, including blood vessels and capillaries that are present as a dense mesh beneath the gastric epithelium. Indeed, *H. pylori* has been detected both close to erythrocytes and within blood vessels in the submucosal surface and lamina propria (Amieva et al., 2003; Semino-Mora et al., 2003; Aspholm et al., 2006; Necchi et al., 2007). Elevated levels of secreted IL-8 and IL-6 (Ding et al., 1997; Innocenti et al., 2002) as well as NF-κB activation (Innocenti et al., 2002) during *H. pylori* infection of endothelial cells have been reported. The molecular details of the *H. pylori*-endothelial cells interplay have however remained under-investigated. A previous study examined the role of *cag*PAI in the induction of inflammatory cytokines in HUVECs but no clear correlation was observed between the *cag*PAI status of *H. pylori* strains and the extent of IL-8 and IL-6 induction (Innocenti et al., 2002).

In this study, we have used a panel of well-characterized isogenic *H. pylori* variants with mutations in the *cag*PAI to unravel the contribution of T4SS and its various key components to IL-8 and IL-6 induction during *H. pylori* infection of endothelial cells. Using primary HUVECs and the HUVEC line EA.Hy926 as models, this study demonstrates for the first time that endothelial cells are significantly more potent than either immortalized or primary gastric epithelial cells in mounting an IL-8 response to *H. pylori* infection (Fischer et al., 2001; Gorrell et al., 2013; Mustapha et al., 2014). However, reminiscent of that observed with *H. pylori-*infected gastric epithelial cells (Gorrell et al., 2013), this potent IL-8 response of *H. pylori*-infected endothelial cells is T4SS- and CagL-dependent. Our findings also show that *H. pylori* T4SS and CagL are essential for IL-6 induction in HUVECs.

Previous studies using co-cultures of *H. pylori* and AGS gastric epithelial cells have suggested that the mechanistic roles of CagL in CagA translocation, elaboration of the T4SS pili and IL-8 induction are tightly coupled (Kwok et al., 2007; Gorrell et al., 2013). One could therefore argue that T4SS component(s) other than CagL is/are the direct stimulus of IL-8 induction and that the role of CagL in IL-8 induction is merely for facilitating the assembly of the T4SS for IL-8 induction. Our data obtained using recombinant CagL protein, however, argue that CagL alone is sufficient for IL-8 induction in HUVECs and that the RGD motif of CagL is crucial for triggering the response. These findings provide strong evidence for a direct and essential role of CagL in IL-8 induction in HUVECs, reminiscent of that observed in *H. pylori* infection of gastric epithelial cells. Nevertheless, the possibility that other components of T4SS function cooperatively with CagL in cytokine induction cannot be discounted.

Our findings suggest that CagA and the CagA translocation process *per se* are not required for IL-8 or IL-6 induction by *H. pylori* during infection of endothelial cells. In line with these observations, a previous study indicated that the *H. pylori* T4SS but not CagA translocation *per se* was involved in stimulating transendothelial migration of T cells (Enarsson et al., 2005). This raises the intriguing possibility that *H. pylori* is unable to translocate CagA into endothelial cells, which is now supported by our findings that neither tyrosyl-phosphorylated CagA nor T4SS-associated pili structures is/are detected in/on *H. pylori*-infected HUVECs. It remains unclear whether this is a HUVEC-specific or cell type-specific phenomenon. Recently, several carcinoembryonic antigen-related cell adhesion molecules (CEACAMs) family members, including CEACAM1, have been shown to interact with the *H. pylori* adhesin HopQ; these interactions are important for CagA translocation into gastric epithelial cells (Javaheri et al., 2016; Koeniger et al., 2016). Given that the inflammatory cytokine TNF-α significantly upregulates CEACAM1 expression in HUVECs (Muenzner et al., 2000), it would be of interest to investigate in future studies whether translocation of CagA occurs in HUVECs after TNF-α stimulation. Moreover, subcellular fractionation should be performed to directly determine whether CagA can be translocated into the cytoplasm of HUVECs.

Our observation that the formation of T4SS-associated pili structures at the *H. pylori*-host cell interface is highly dependent on the host cell type is equally interesting. This is consistent with the notion that these appendages are assembled in response to highly specific host cell signals. Our findings that no such pili are visible at the interface of *H. pylori* and HUVECs suggest a non-essential role of these pili for IL-8 or IL-6 induction in *H. pylori*-infected HUVECs. However, one cannot rule out the possibility that T4SS pili are still formed albeit as rather inconspicuous structures that escape detection by scanning electron microscopy. A comparison of the interacting partners of the *H. pylori* T4SS in HUVECs with those in AGS cells may provide novel insights into the molecular mechanisms behind CagA translocation and the formation of the T4SS pili.

**Figure 8:**
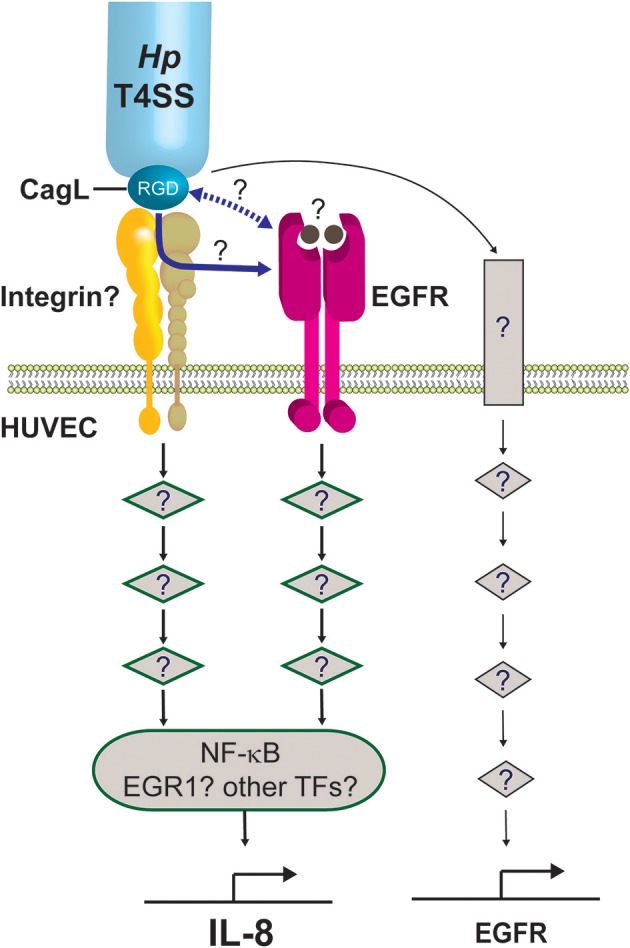
Working model on the molecular mechanism by which the *H. pylori* T4SS induces potent IL-8 induction in human endothelial cells. We propose that the *H. pylori* T4SS triggers IL-8 induction in endothelial cells by interacting with an as yet unidentified integrin (shown as yellow/beige heterodimer) via the T4SS-associated adhesin, CagL, and its RGD motif. This triggers the activation of downstream signaling intermediates (diamond symbols), leading to activation of NF-κB and subsequent expression of IL-8. Other transcription factors (TFs) important for regulating inflammatory genes, such as EGR1, AP-1, or STAT3, may also contribute to the induction of IL-8 in endothelial cells. This study shows that the *H. pylori* T4SS/CagL is capable of upregulating EGR1 expression (not depicted). The latter is a known downstream response of EGFR activation. The precise mechanism by which the *H. pylori* T4SS/CagL triggers activation of EGFR signaling is currently unclear. It may occur either via integrin-EGFR crosstalk subsequent to the binding of CagL to integrin (blue solid arrow) or direct interaction of CagL with EGFR via an unknown mechanism (blue dashed arrow). Alternatively, CagL-integrin interaction may activate an ADAM17-like metalloprotease that results in the shedding of heparin-binding epidermal growth factor (HB-EGF) (solid circles), which then binds to EGFR and activates EGFR signaling. Finally, our findings show that *H. pylori* T4SS/CagL is able to upregulate EGFR expression. We propose that this occurs via stimulation of a hitherto unknown receptor (rectangle) and the corresponding downstream signaling pathway, acting as a positive feedback mechanism by which *H. pylori* further potentiates EGFR-dependent IL-8 induction in HUVECs. The number of signaling intermediates illustrated is arbitrary.

This study shows that whilst integrins α_5_β_1_ and α_v_β_3_ are dispensable for IL-8 induction by *H. pylori* in endothelial cells, EGFR plays a major role in the response. Given the essential requirement for CagL and its RGD motif in IL-8 induction, it is however tempting to speculate that an as yet unidentified integrin member might be involved in the binding of CagL. It is plausible that this integrin may transduce the downstream signals either independently of EGFR or via crosstalk with EGFR in mediating the proinflammatory response (Figure 8).

Transactivation of EGFR by *H. pylori* induces upregulation of the transcription factor, EGR1, in the gastric epithelial cell line AGS (Keates et al., 2005). EGR1 regulates many cellular processes including proliferation, angiogenesis and inflammatory responses (Thiel and Cibelli, 2002), and its role as a downstream effector of EGFR is well-established (Cabodi et al., 2009). Whilst the involvement of *cag*PAI in EGR1 upregulation has been described for *H. pylori* infection of AGS cells (Keates et al., 2005), this is the first report of *cag*PAI and CagL playing a role in EGR1 upregulation in *H. pylori*-infected HUVECs. EGR1 is known to function synergistically with NF-κB in upregulating IL-8 production in prostate cancer cells (Ma et al., 2009), and that its expression can be regulated by EGFR-integrin crosstalk (Cabodi et al., 2009). Thus, it is tempting to speculate that EGR1 might play a role in IL-8 induction in *H. pylori*-infected endothelial cells via a CagL/EGFR/integrin/EGR1/NF-κB nexus (Figure 8). In line with this model, our results show that inhibition of NF-κB activation in *H. pylori*-infected HUVECs only partially suppresses IL-8 induction. The relative contribution of EGR1, NF-κB, and possibly also other transcriptional factors (e.g., AP-1, STAT3) to the inflammatory response, however, remains to be determined. Meanwhile, our findings indicate that *H. pylori* is capable of upregulating EGFR expression in HUVECs in a manner dependent on CagL and *cag*PAI, which may act as a positive feedback mechanism by which *H. pylori* further potentiates EGFR-dependent IL-8/6 induction in HUVECs (Figure 8). Taken together, the detailed molecular mechanism by which *H. pylori* T4SS and CagL activate RGD-motif-dependent and EGFR-dependent IL-8 induction is likely to be intricate, and is the subject of ongoing investigations in our laboratories.

IL-8 plays a pivotal role in inflammation, angiogenesis and cancer. By examining a previously unappreciated role of endothelial cells in eliciting a strong IL-8 response against *H. pylori* infection, this study provides important new insights into the pathogenesis of *H. pylori*-associated gastric diseases. In particular, our results support the notion that apart from gastric epithelial cells, endothelial cells may also contribute to *H. pylori*-induced inflammation. This study also reveals for the first time that the T4SS-associated adhesin, CagL, and the tyrosine kinase receptor EGFR play key roles in IL-8/IL-6 induction during *H. pylori* infection of endothelial cells; this not only pinpoints a novel mechanism by which the *H. pylori* T4SS may contribute to chronic inflammation but also highlights the importance of CagL and EGFR as therapeutic targets for treatment against chronic gastritis and gastric cancer. Future work is required to further understand the molecular mechanism by which *H. pylori* activates EGFR in endothelial cells, the effect of endothelial cell polarity on IL-8/IL-6 induction, and the relative contribution of endothelial inflammatory signaling to gastric carcinogenesis.

## Author contributions

MT, RG, RD, BJ, ED, and TK: designed the experiments; MT, JG, YX, VD, NC, RG, TK, and MR: generated experimental data; MT, JG, YX, RG, VD, NC, MR, BJ, ED, and TK: analyzed experimental data; RP, RD, and BJ: provided bacterial strains and reagents; MT, TK, and RG: wrote the manuscript and prepared the figures; JG, YX, VD, MR, RP, BJ, and ED: critically reviewed the manuscript.

### Conflict of interest statement

The authors declare that the research was conducted in the absence of any commercial or financial relationships that could be construed as a potential conflict of interest.
