# Supplementary material for: Helicobacter pylori Type IV Secretion System and Its Adhesin Subunit, CagL, Mediate Potent Inflammatory Responses in Primary Human Endothelial Cells
Source: Front Cell Infect Microbiol. 2018 Feb 6;8:22. doi: 10.3389/fcimb.2018.00022 (PMC5808116; doi:10.3389/fcimb.2018.00022)
Supplement: Supplementary file 1 [file Image1.PDF]

Supplementary Material

Helicobacter pylori CagL mediates potent inflammatory responses in primary human endothelial cells

Mona A. Tafreshi, Jye S. Guan, Rebecca J Gorrell, Nicole Chew, Yue Xin, Virginie Deswaerte, Manfred Rohde, Roger J. Daly, Richard M. Peek, Jr., Brendan Jenkins, Elizabeth M. Davies, Terry Kwok*

*** Correspondence:** Terry Kwok: terry.kwok@monash.edu

Supplementary Figures

Supplementary Figure 1. Level of adherence of wild-type *H. pylori* P12 and various isogenic mutants to HUVECs. 2

Supplementary Figure 2. IL-8 induction by *H. pylori* 7.13 upon infection of AGS or HUVECs is CagA-independent. 3

Supplementary Figure 3. Interaction of *H. pylori* with HUVECs does not result in CagA translocation. 4

Supplementary Figure 4. The integrin function-blocking antibodies blocked cell attachment to extracellular matrix proteins. 5

Supplementary Figure 5. The role of EGFR in IL-6 induction by *H. pylori* upon infection of HUVECs. 6

Supplementary Figure 6. Treatment of HUVECs with AG1478 at concentrations up to 20 μM did not reduce cell viability. 7

Supplementary Figure 7. IκB kinase inhibitor BMS345541 inhibits *H. pylori*-induced NF-κB activation in human primary endothelial cells. 8

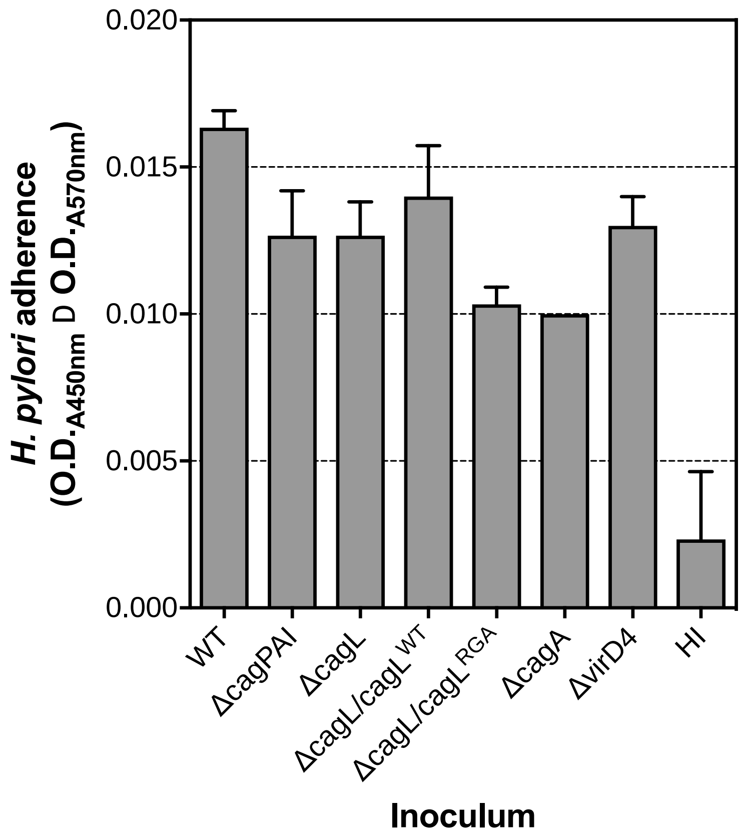

Supplementary Figure 1. Level of adherence of wild-type *H. pylori* P12 and various isogenic mutants to HUVECs.

HUVECs were infected with H. pylori P12 WT and various isogenic mutants at MOI = 10. The mean level of H. pylori adherence to HUVECs at 24 hpi was determined by colorimetric detection of immunolabeled bacteria in a bacterial attachment assay. Error bars denote SD of triplicate samples from a single experiment.

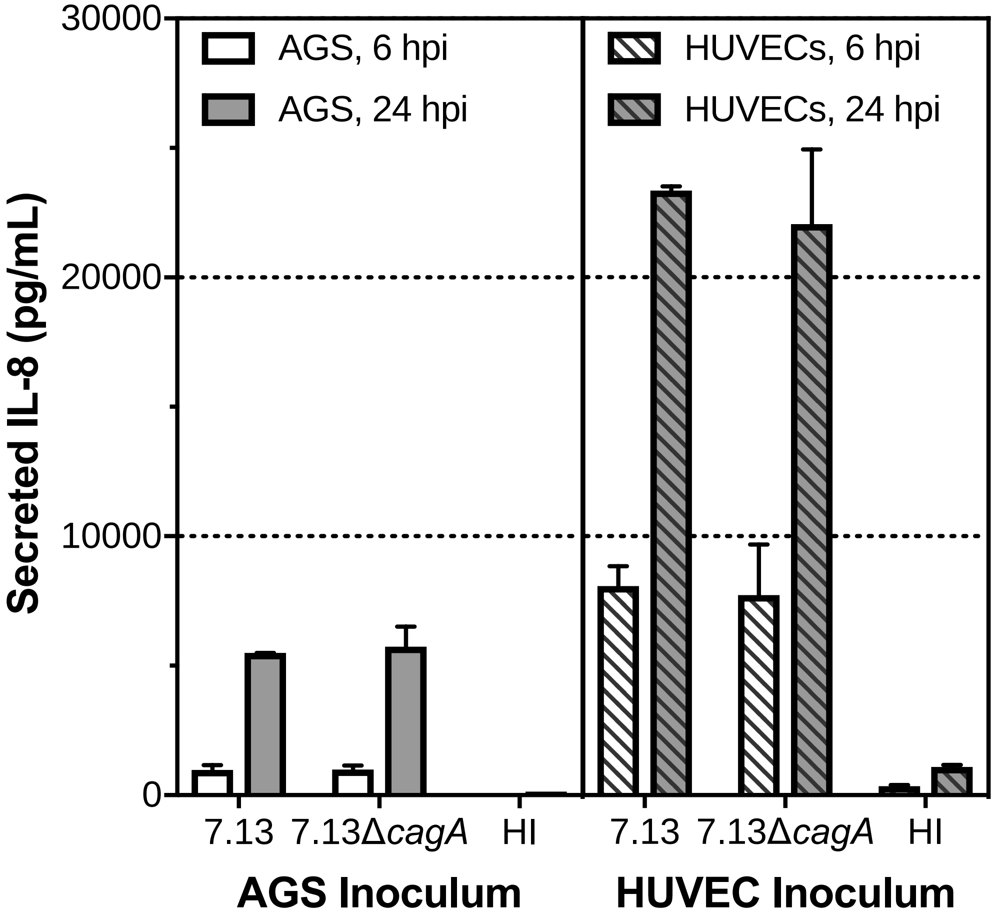

Supplementary Figure 2. IL-8 induction by *H. pylori* 7.13 upon infection of AGS or HUVECs is CagA-independent.
AGS cells were infected with *H. pylori* 7.13 wild-type (WT) or isogenic *ΔcagA* mutant at MOI = 100; HUVECs were similarly infected with *H. pylori* 7.13 wild-type (WT) or isogenic *ΔcagA* mutant, but at MOI = 1. Mean IL-8 secretion was determined by ELISA in spent culture media harvested at 6 hpi or 24 hpi. Error bars denote SD; N=2.

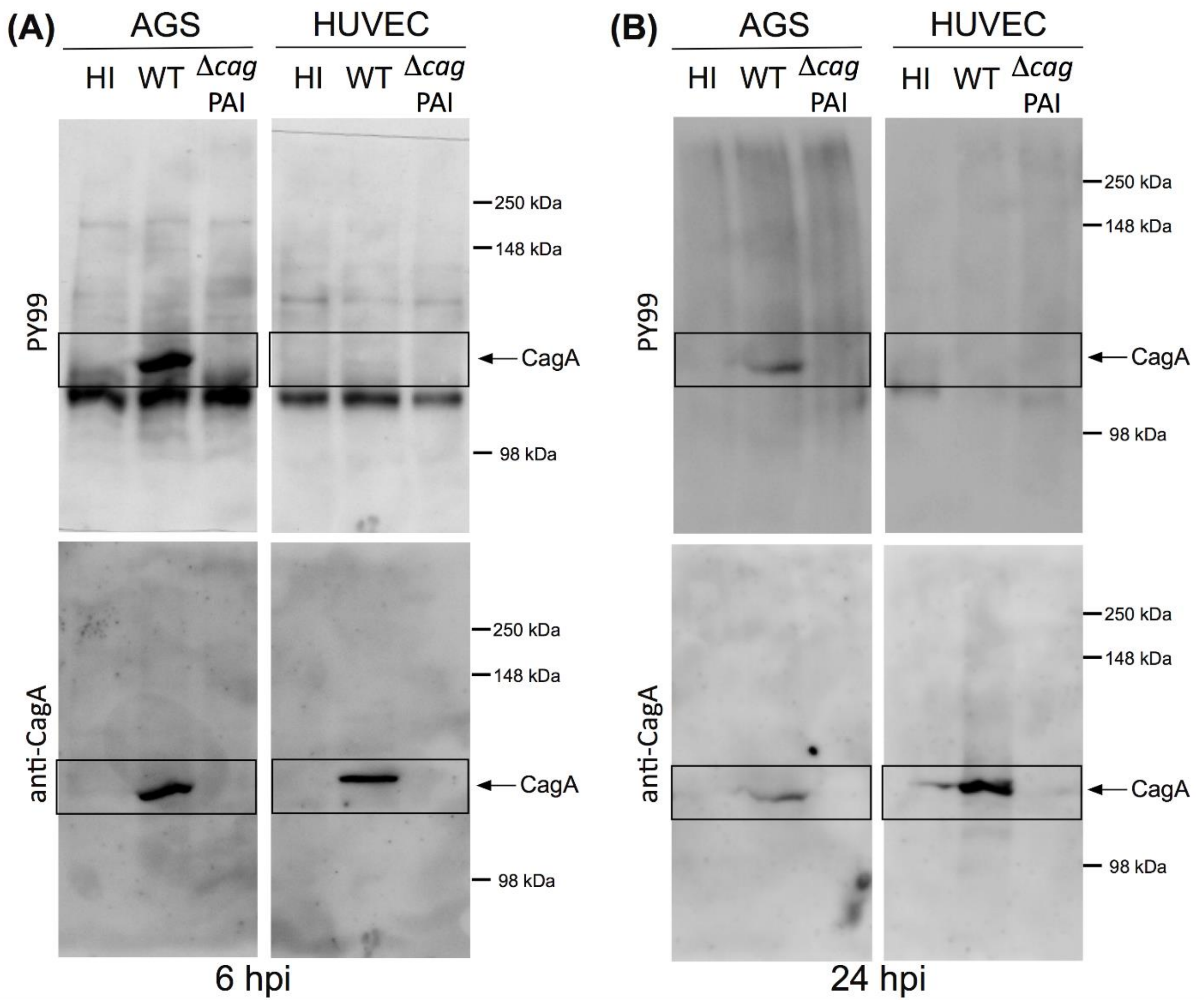

Supplementary Figure 3. Interaction of *H. pylori* with HUVECs does not result in CagA translocation.
Complete images of the blots shown in Fig.4. Rectangles indicate the selected areas of interest that are shown in Fig. 4.

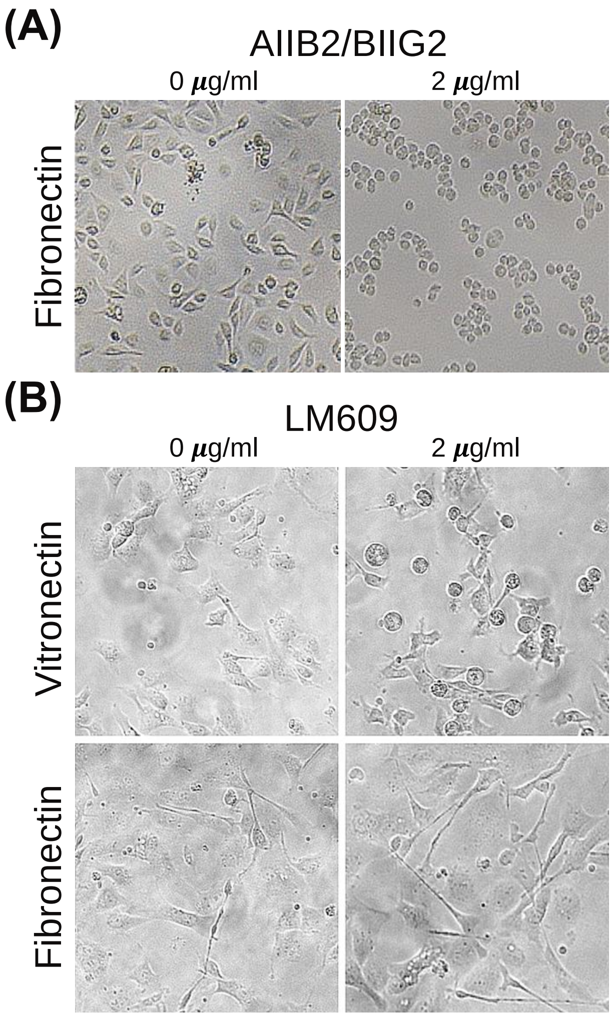

Supplementary Figure 4. The integrin function-blocking antibodies blocked cell attachment to extracellular matrix proteins.
(A) AGS pre-treated with PBS (0 µg/ml), or integrin $\alpha_5\beta_1$ function-blocking antibodies AIIB2 (2 µg/ml) and BIIG2 (2 µg/ml) in combination, were allowed to attach to wells coated with 100 µg/ml fibronectin (specific ligand for $\alpha_5\beta_1$). **(B)** HUVECs pre-treated with PBS (0 µg/ml, negative control), or integrin $\alpha_v\beta_3$ function-blocking antibody LM609 (2 µg/ml), were allowed to attach to wells coated with 100 µg/ml vitronectin (specific ligand for $\alpha_v\beta_3$) or fibronectin. Cells whose spreading was inhibited appeared rounded.

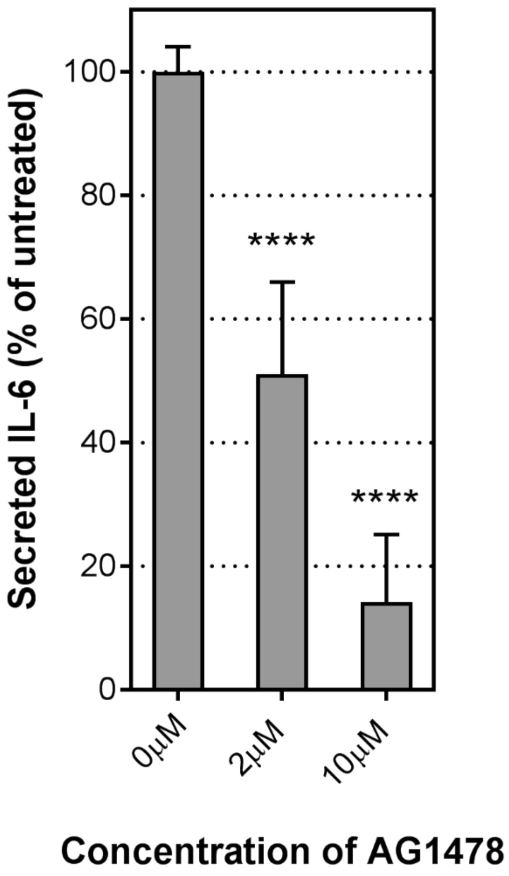

Supplementary Figure 5. The role of EGFR in IL-6 induction by *H. pylori* upon infection of HUVECs.
Prior to incubation with *H. pylori* P12 (MOI =1), HUVECs were pre-treated with the EGFR small molecule inhibitor AG1478 at various concentrations indicated. Spent culture media was harvested at 24 hpi and assayed by ELISA for secreted IL-6. IL-6 levels are expressed as the mean percentage of that determined for P12 WT-infected HUVECs without pre-treatment with AG1478 (untreated). Error bars denote SD, N=2; statistical analysis of AG1478 dose-response by two-way ANOVA (Tukey's multiple comparisons post-test); significant differences compared to 0 µM are shown; ****, $p<0.0001$.

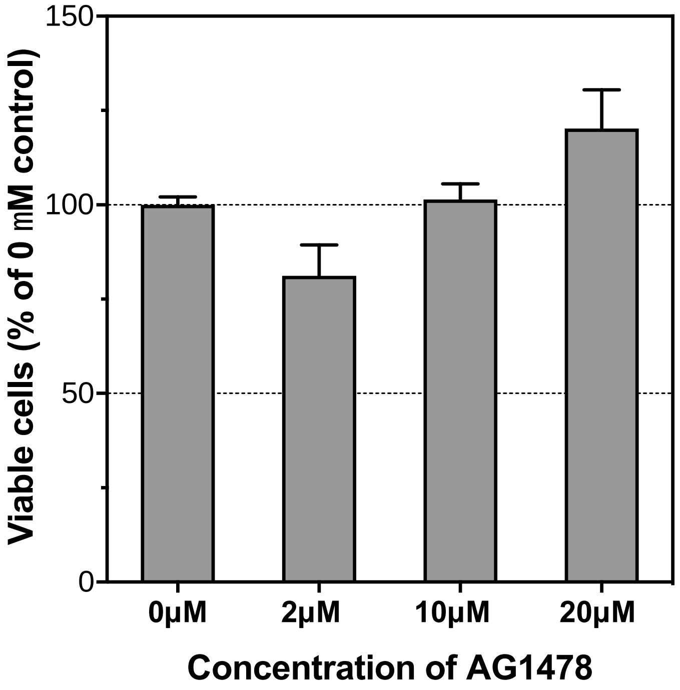

Supplementary Figure 6. Treatment of HUVECs with AG1478 at concentrations up to 20 µM did not reduce cell viability.

HUVECs were incubated with AG1478 at concentrations 0.5, 2, 10 or 20 µM for 24h. Cells were then trypsinized and diluted in 0.4% (w/v) trypan blue in PBS (pH 7.2) for enumeration of viable and non-viable cells. Viability is expressed as the mean percentage of that determined for untreated (0 µM AG1478) HUVECs. Error bars denote SD; N=2.

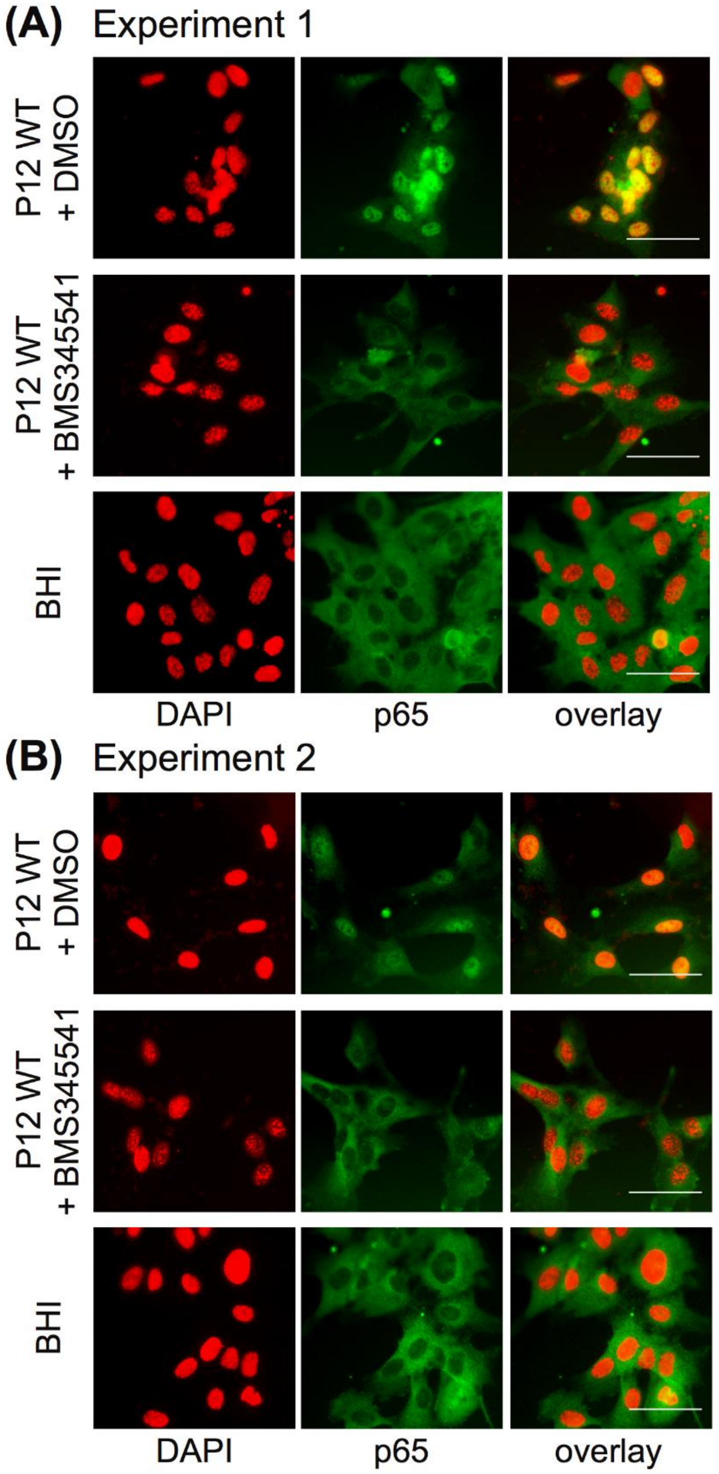

Supplementary Figure 7. IκB kinase inhibitor BMS345541 inhibits *H. pylori*-induced NF-κB activation in human primary endothelial cells.
HUVECs inoculated with *H. pylori* P12 (MOI of 50) following pre-treatment of the cells with DMSO or IκB kinase inhibitor BMS345541 (10 µM), or inoculated with sterile BHI broth, were fixed with PFA at 3 hpi. Fixed cells were stained for NF-κB p65 (green) and nuclei were counterstained with DAPI (red); yellow/orange in merged images denotes nuclear p65. Scale bar, 50 µm. Images from 2 independent experiments are shown.
